# Progress of iPSC-derived retinal organoids in the study of inherited retinal diseases

**DOI:** 10.1186/s13023-025-04059-7

**Published:** 2025-10-31

**Authors:** Bochen Liu, Yi Liu, Haiyan Zhang, Jing Huang, Guohua Liu

**Affiliations:** 1 Pediatric Research Institute, Children’s Hospital Affiliated to Shandong University (Jinan Children’s Hospital), Jinan, China; 2 Department of Ophthalmology, Children’s Hospital Affiliated to Shandong University (Jinan Children’s Hospital), Jinan, China; 3 Shandong Provincial Clinical Research Center for Children’s Health and Disease, Jinan, China

**Keywords:** Induced pluripotent stem cell, Retina, Retinal organoids, Inherited retinal diseases, Retinoblastoma, Retinitis pigmentosa, Leber congenital amaurosis, X-linked juvenile retinoschisis

## Abstract

Inherited retinal diseases (IRDs) constitute a complex and heterogeneous group of rare disorders characterized by significant genetic diversity. These conditions often lead to the degeneration of photoreceptor cells, resulting in severe visual impairment. A major challenge in the study and treatment of IRDs is the lack of appropriate preclinical models for investigating their pathogenesis and evaluating potential therapeutic interventions. In recent years, advances in retinal organoids (ROs) culture technology have provided promising new avenues for IRDs. This review systematically elaborates on the applications of induced pluripotent stem cells (iPSC)-derived ROs as disease models in IRDs such as retinoblastoma (RB), retinitis pigmentosa (RP), Leber congenital amaurosis (LCA), and X-linked juvenile retinoschisis (XLRS). This review also briefly explores the culturing methods of iPSC-derived ROs in recent years. Specifically, it emphasizes the comparison of the similarities between the formation process of ROs and the in-vivo retinal development process. This encompasses aspects such as the formation of different structures, the expression of key markers, and the manifestation of physiological functions. By utilizing the RO disease model to explore the pathogenesis of IRDs and conduct drug screening, it is expected to advance the precision treatment of IRDs and improve the therapeutic outcomes for patients.

## Introduction

Inherited retinal diseases (IRDs) are a heterogeneous class of monogenic retinal disorders that are associated with more than 280 disease-causing genes. The prevalence of IRDs is approximately 1 in 1,380, and it is predicted that 5.5 million individuals globally will be impacted [1, 2]. These diseases are characterized by mutations in diverse retinal cell types, including retinoblastoma (RB), retinitis pigmentosa (RP), Leber congenital amaurosis (LCA), and X-linked juvenile retinoschisis (XLRS) [3]. IRDs represent a diverse group of visually debilitating diseases that can cause blindness from birth/early infancy or over time where disease-causing variants in genes critical to retinal structure and/or function lead to photoreceptor cell dysfunction/death and associated vision loss [4]. Although individuals with IRDs are classified as having rare diseases, IRDs still represent the largest group of untreatable diseases in ophthalmology [5]. Since IRDs can lead to progressive vision loss, they not only have a negative impact on patients’ physical health and their families, but also imposes significant social costs, including loss of individual productivity, costs associated with the provision of health care, and deadweight loss to society [3].

In the 1970s, the two-dimensional (2D) cell model has been widely applied for in vitro culture [6]. However, it also brings a series of problems. This is because it is difficult for 2D cells to reconstruct the true microenvironment of cells, such as loss of their surface markers and alteration of natural cell morphology. Meanwhile, the animal model also has limitations, and there are species differences from human which makes it challenging to extrapolate the generated results [7]. To overcome the limitations of these experimental models, Hans Clever successfully established the first 3D organoid culture system in vitro in 2009, ushering in the era of organoids as disease models [8]. Since then, various types of organoids, such as liver organoids, kidney organoids, heart organoids, retinal organoids, etc. have also been cultured [7]. Organoids, derived from the body’s own tissues or stem cells, are 3D structural models of original tissues and organs formed by 3D culture in vitro. Organoids are highly similar to source tissues in terms of tissue structure, cell type, and function, and they are becoming key tools in disease modeling, pharmacodynamic research, and precision medicine [9]. Organoids have two important characteristics: Firstly, organoids induced by stem cell differentiation should have the ability for self-renewal; secondly, these cells have the ability to self-assemble and develop into organoid tissue [10]. In addition, these organoids are capable of sustaining self-renewal and self-assembly over the long term.

Organoids are constructed in a variety of ways. Organoids derived from stem cells include induced iPSCs, embryonic stem cells (ESCs), and organ-restricted adult stem cells (ASCs), which can be constructed only by information carried by the cell itself or induced by the scaffold system [11]. The establishment of ASC organoids relies on the support of a mixture of growth factors that mimic normal stem cell homeostasis factors in the corresponding medium. The establishment of pluripotent stem cell (PSC) organoids is achieved by continuously adding a specific combination of growth factors to simulate cell signaling during embryonic development, thereby driving the multi-step expansion and differentiation of PSCs [12].

Despite significant advances in the treatment of IRDs, it remains a major health problem globally that needs to be addressed [13]. The main problem in studying IRDs is the lack of suitable preclinical models, which makes it difficult to move from basic research findings to translational clinical treatment strategies. In recent years, through regenerative medicine and stem cell therapy, iPSCs can be induced to differentiate in vitro and cultured into 3D structured ROs [14]. ROs maintain the layered structure of the retina and reproduces the process of retinal development in vivo, which is of great significance for the study of human retinal development and the establishment of retinal disease models in specific genetic contexts [15].

This article aims to systematically integrate and critically analyze recent advances in research on iPSC-derived ROs in the context of IRDs. It analyzes various protocols for the induction and differentiation of ROs, while also summarizing the key stages of in vivo retinal development. Particular emphasis is placed on comparing the structural and physiological functional similarities between ROs and native retinal development in vivo. By synthesizing findings from multiple studies, this review seeks to provide novel insights that may inform both basic research and potential therapeutic strategies for IRDs.

## RO development: recapitulating in vivo retinogenesis

### The structure and development of the retina

During early embryonic development, the retina is formed from the neuroectoderm (the outermost embryonic germ layer). The ectoderm forms two optic vesicles (OVs), the distal portion of which folds inward to form the optic cup. The outer and inner walls of the optic cup produce RPE and retina, respectively [16]. The origin of mammalian neuroretinal cells is a continuous but overlapping process. The development process is divided into two stages: early (7 to 18 weeks) and late (18 to 23 weeks) [17]. In the early stage, retinal ganglion cells, cone photoreceptors, amacrine cells and horizontal cells differentiate successively [18]. In the late stage, bipolar cells and Müller glial cells born [19]. The development of photoreceptors is from early to late in fetal retinal development. By 14 weeks, the human retina has formed three cellular layers of the neuroretina and two synaptic layers. At 20 weeks, the normal human central retina contains a population of mature retinal cells from all seven retinal cell types [20].

The retina detects visual information from the outside world and converts these light signals into chemical signals that are transmitted to the cerebral cortex for imaging [21]. The retina consists of the neural retina (NR) and a non-neural supporting layer of retinal pigment epithelium (RPE). The NR is a stratified structure made up of 55 different cell types, including various types of bipolar cells, horizontal cells, numerous types of amacrine cells, Müller glia, and photoreceptors (cones and rods), and retinal ganglion cells (RGCs) [22]. The RPE is a single layer of arranged hexagonal cells that can support and nourish photoreceptor cells, block light, dissipate heat, regenerate and repair. The NR is composed of three retinal nuclear layers (laminae) and two intermediary synaptic plexiform layers. Electrochemical signals are processed sequentially in these layers prior to being transmitted to the brain [16].

When light reaches the retina, rod and cone photoreceptors fulfill a photosensitive function and initiate a phototransduction cascade. The graded membrane potential is generated in the outer nuclear layer (ONL), where rod and cone photoreceptors reside, and is subsequently transmitted and processed in the outer plexiform layer (OPL) through synaptic connections with neurons in the inner nuclear layer (INL). INL consists of cell bodies of bipolar, horizontal, apodal, and interplexal cells that regulate local signal processing in the retina. RGCs are the “output” neurons of the retina. The RGC receives signals from bipolar and aphotic cells, integrates and encodes visual information from the surrounding receptive field, and generates action potentials that travel to the optic nerve and are eventually transmitted to the cerebral cortex for imaging [23].

### The differentiation process of ROs

Retinal organoid differentiation is similar to the formation of OVs. After long-term cultivation using existing culture protocols, RO can contain most retinal neuron cell types, such as rod and cone cells, ganglion cells, horizontal cells, bipolar cells, and Müller cells, and can mimic the stratigraphy of the retina [24, 25].

The differentiation of RO can be broadly categorized into three distinct stages [26]. In the initial stage, occurring between differentiation days (D) 30 and 50, the organoids exhibit a well-defined and luminous outer neuroepithelial margin populated by neuroretinal progenitor cells (NPCs) and RGCs. RGCs are the first retinal cell type to undergo differentiation, with their numbers declining after D90 [24]. The second stage spans approximately from D80 to D120, during which the organoids develop a dark-phase core; concurrently, the bright margins diminish as early progenitors of cone and rod photoreceptors begin to emerge. In the final stage, occurring around D120 to D180, there is an enhancement in the prominence of the outer edge while photoreceptor structures commence their differentiation process [27].

RO recapitulates the 3D histoarchitecture and functionality of the human retina [28]. Studies have shown that RO can simulate the early stages of human eye development and the onset of human retinal diseases, and the layer structure can display human-specific characteristics, including an outer nuclear layer with photoreceptors (rhodopsin+, recoverin+) (CRALBP+), and a RGC layer with RGCs (Brn3+, Pax6+, calretinin+) and among others [29]. Another important characteristic of RO is variety of photoreceptors. By electron microscopy imaging, there are other ultrastructural features of mature photoreceptors in RO, including an outer limiting membrane, basal bodies with connecting cilia displaying a photoreceptor-specific microtubule arrangement, and so on [30, 31]. In addition, the most typical feature of RO is its ability for phototransduction, the specific process is: the receptor plays a photosensitive role, triggers a cascade reaction, and causes the hyperpolarization of the cell membrane potential at the synapse, so that the light signal is transformed into an electrical signal to continue to transmit. Existing studies have shown that iPSC-induced ROs has detected the expression of photoreceptors and essential proteins involved in photoconduction, such as opsin and rhodopsin [32].

### The phototransduction of ROs

ROs incorporate all major retinal cell types. They recapitulate the in vivo retinogenesis process of the “forebrain - optic vesicle - optic cup” sequence and display a laminar structure analogous to that witnessed in native retinas [33]. Furthermore, ROs share comparable physiological functions with in - vivo retinas. They are capable of replicating the phototransduction cascade and synaptic connections. As a tool for investigating retinal development and diseases, ROs must exhibit several specific characteristics to elicit functional photoreactions. Specifically, the properties of photoreceptor cell membranes should be favorable for signal propagation; the phototransduction cascade must be established and functioning properly; and synaptic connections need to be achieved [34]. Photoreceptors possess numerous ion-membrane channels, which serve to maintain their membrane potential and regulate phototransduction signals [35]. These ion-membrane channels are of utmost importance during the phototransduction process. The whole-cell patch clamp technique can be used to detect the function of photoreceptor membrane channels in ROs. Saha et al. verified the existence of mature and functional cyclic nucleotide - gated (CNG) and hyperpolarization-activated cyclic nucleotide-regulated (HCN) channels in photoreceptors, which indicates the appropriate intracellular mechanisms for light responses. Meanwhile, it is further demonstrated that cone cells derived from ROs elicit a strong, graded, reproducible, and wavelength-specific light-evoked electrophysiological response (i.e., phototransduction) [36]. Calcium imaging can be employed to visualize the light responses originating from photoreceptors and the synaptically driven responses of cells within the inner nuclear layer and ganglion cell layer. Cowan et al. reported that there were corresponding reproducible responses in certain cells of the photoreceptor layer, inner nuclear layer, and ganglion cell layer [24].

## Derivation and culture of iPSC-ROs

Here, we focus on the culture system of iPSC-derived RO. Early studies have shown that suspension cultured human iPSCs can be successfully induced into heterogeneous cultured cells expressing retinal progenitor cell and RPE cell markers by utilizing Wnt and Nodal antagonists [3]. These signal transduction pathways have previously been studied as important regulatory molecules in retinal development [37]. Later, the researchers found that when these heterogeneous cultures were treated with retinoic acid and taurine, the expression of photoreceptor cell markers was detected. The inhibition of Notch signaling by DAPT treatment significantly increased the proportion of photoreceptor cells and RPE cells [37, 38]. A neuro-inducing medium enriched with heparin and a chemically defined N2 supplement facilitates the aggregation of iPSCs into embryoid bodies (EB), which subsequently adhere to the surface of the petri dish and differentiate into NR [39]. Therefore, the culture program for inducing iPSC to differentiate into RO is gradually improved and perfected to enhance the maturity of RO [40].

### The 3D and a combination of 2D/3D culture model

Past RO culture systems are mainly categorized into two types: 3D, and a combination of 2D and 3D [20, 41]. The key difference lies in the EB stage. The 3D culture system commences with the EB stage, followed by the OV and optic cup stages. However, the 2D/3D culture system bypasses the EB stage and generates the OV stage from adherent retinal cells [42]. In 2011, Meyer et al. demonstrated that iPSCs can differentiate into 3D structures resembling OV, which include photoreceptors and RPE cells; these structures exhibit electrophysiological properties characteristic of phototransduction [43]. Subsequently, in 2014 Zhong et al. employed a combination of 2D and 3D cultures to circumvent embryoid formation, thereby generating neuroretinal structures in an adherent culture system, which were subsequently excised and further cultured in suspension [44]. Specifically, the culture process initially employs conditioned medium to facilitate the development of 3D retinal cups. Subsequently, during further differentiation, the retinal cups are manually dissected from the adjacent RPE cells. Additionally, taurine and retinoic acid are introduced at later stages to support the long-term maintenance of ROs. This protocol yielded ROs with functional photoreceptors exhibiting outer segment discs and light sensitivity [45]. Subsequently, some researchers further optimized the cultivation conditions, aiming to obtain higher RO production efficiency. For example，Kuwahara et al. developed an optimal culture method for breedless hPSCs: preconditioning the initial hPSC state by modulating the TGF-β and Sonic hedgehog signaling pathways, applying SAG on day zero, and timing the introduction of BMP4 [46]. Additionally，9-cis-retinal has been shown to enhance differentiation of photoreceptor cells while supplementation with BMP4 and IGF-1 significantly promotes OV formation as well as lamination [27].

To meet the need for clinical transformation, Reichman et al. developed a simple method for generating storable ROs using completely defined xeno-free and feeder-free conditions [47]. The protocol minimizes the use of exogenous differentiation factors by relying on iPSC-induced endogenous Dickkopf-associated protein 1 (DKK1) and Noggin, which are essential for the self-formation of retinal structures [46]. This methodology has the potential to induce retinal progenitor cells (RPCs) to spontaneously differentiate into various cell types, faithfully summarizing the complex spatio-temporal pattern of retinal development. By day 100, retinal organoids contain almost all retinal cell types, including mature photoreceptors, bipolar cells, horizontal cells, amacrine cells, and Müller glial cells.

### Advantages and challenges

Compared with the simple 3D culture model, the combination of 2D and 3D culturing methods generally allows for more effective organization and maturation into advanced organoids presenting a layered retinal morphology [3]. It is feasible to recapitulate each major step of retinal development observed in vivo in a highly autonomous manner in both spatial and temporal dimensions. The derived neuro-retinal vesicles feature a well-defined ONL-like structure. This structure contains cones and rods equipped with inner segments and connecting cilia, nascent outer segments, and presynaptic structures. This differentiation system recapitulates the developmental process of human photoreceptors, thereby enabling the isolation and transplantation of a pure population of developmentally matched cone cells. Subsequently, the 3D retinal cups gradually take shape, encompassing all major retinal cell types arranged in proper hierarchical order [31, 44].

It is important to note that the meticulous separation of OV structures is crucial for the differentiation and maturation of ROs. Capowski et al. employed an ophthalmic scalpel to meticulously dissect the OV-like region, while Sanjurjo-Soriano et al. utilized scalpel resection to accurately identify and excise neuroretina-like structures in their study [27, 48]. Nevertheless, the intricate dissection of optic vesicular structures remains a labor-intensive and time-consuming endeavor that necessitates extensive practice and training [49]. Regent et al. describe a simple and effective method for isolating visual vesicle-like domains from adherent cell cultures that is simpler than dissection and provides better results for the production of retinal organoids [50]. Their method involved using a cell scraper to scrape off the entire adherent culture and then growing the cell aggregates in free-floating conditions. This approach eliminates the need for subjective selection of visual vesicle-like domains and provide an alternative approach to improve the efficiency of RO differentiation.

## iPSC-ROs in IRD modeling

Building a 3D organoid disease model of the retina can better simulate the interaction between tissue cells and stroma, cell migration, etc., and provide a promising tool for exploring disease mechanisms and evaluating potential therapies for currently incurable retinal diseases. IRD, one of the most common genetic disorders in humans, defines a group of clinically heterogeneous diseases that result in vision loss due to maldevelopment, dysfunction, or premature death of photoreceptor cells in the retina. IRD is characterized by several factors, including the type and location of affected cells and the timing of the onset of the disease. Common IRDs include retinitis pigmentosa, retinoblastoma, Leber congenital amaurosis, X-linked juvenile retinoschisis, and so on [51]. Human PSC-derived RO models are potent tools for identifying disease mechanisms and developing novel therapeutics. To date, over two dozen studies have utilized patient-derived or gene-edited human PSC-derived ROs to model IRDs [42]. This offers a good in vitro model for investigating the pathogenesis and treatment strategies of IRDs.

### Retinitis pigmentosa

RP is a genetically determined retinal disease characterized by the loss of photoreceptors and the presence of retinitis pigmentosa, which is accompanied by early-onset nocturnal vision, followed by progressive degeneration of photoreceptors, resulting in irreversible vision loss [52]. RP is the most common IRD, with an incidence of approximately 1 in 4000 [53]. It can be either autosomal recessive (50–60% of patients), autosomal dominant (30–40% of patients), or X-linked (5–15% of patients) [54]. Currently, around 70 gene mutations are known to trigger the onset of RP, including *USH2A*, *RPGR*, *RHO*, and others. In most cases, vision loss in RP patients is due to degeneration of photoreceptor cells or RPE cells caused by genetic mutations. Because the underlying mechanisms of cell death are largely unknown, few effective treatments have been developed.

USH2A mutations are the predominant cause of non-syndromic RP. Studies have indicated that reprogramming-induced iPSCs from RP patients with USH2A mutations lead to the cultivation of multilayered retinal organoids consisting of NR and RPE. Early retinal organoids derived from patients with RP due to USH2A mutations show significant deficiencies in morphology, immunofluorescence staining, and transcription profiles [55]. This abnormal retinal neuroepithelial differentiation and polarization leads to defects in RPCs development and retinal layer formation, and NR tissue disorder in the presence of USH2A mutations. In addition, the expression of cilia-related genes and dopaminergic synaptic genes in USH2A mutated organoids was low. However, the expression of genes related to neuronal apoptosis was higher. This work outlines the pathogenesis of USH2A using patient-specific organoids and demonstrates that mutation-induced alterations in USH2A function can lead to cellular and molecular abnormalities.

Patient-specific ROs with RHO mutations are the main cause of autosomal dominant RP, which is manifested by disrupted photoreceptor development, increased rod cell apoptosis induced by ER stress, and progressive loss of rod photoreceptors [56]. In the long-term culture of organoids, photoreceptor cells were found to be underdeveloped, RHO mRNA expression was increased, and rhodopsin protein was incorrectly localized in rod photoreceptor cells [57].

In addition, ROs mutations in other genes, such as *PRPF31*, *CRB1*, *RPGR*, and others, have also summarized the pathogenesis of this gene; these studies may provide important help for molecular diagnosis and screening of different types of RP.

### Retinoblastoma

Rb is the most common intraocular malignancy among children. Most cases are diagnosed before the age of 5 and have a poor prognosis, with a global survival rate of less than 30% [58]. Mutations in the RB1 gene are the sole cause of inherited retinoblastoma, and the protein it encodes is a key regulator of the cell cycle [59]. Biallelic inactivation of the RB1 gene is a causative factor in 96% of cases of this retinal malignancy, resulting in the formation of a non-invasive tumor known as a retinoma [60]. Despite numerous studies, it remains unclear why retinal tissue is selectively prone to malignant transformation in individuals with heterozygous germline mutations in RB1.

By using iPSC-induced ROs from patient-derived *RB1* mutations as a disease model, the study demonstrated that pRB-depleted retinal organoids displayed characteristics resembling those of Rb tumors, including mitochondrial ridge aberrations and rose-like structures, and were capable of cell transformation in vitro. This could contribute to better characterizing these tumors and might have potential therapeutic significance. The application of Topotecan and TW-37 led to a significant reduction in the proportion of Rb proliferative cone precursors, confirming the suitability of these in vitro models for testing new therapies for Rb [61]. Gene editing of pluripotent stem cells to induce their progressive differentiation into Rb organoids can successfully observe tumorigenesis in retinal organoids, reveal high-fidelity genetic and epigenetic characteristics, map the origin of cancer cells, and test potential therapeutic drugs [62]. Single-cell RNA sequencing of RB-RO cells demonstrated that retinal progenitor cells constituted the most prevalent cell type. Furthermore, the sequencing outcomes were unable to differentiate between RB-RO derived from RB patients and xenografts originating from in situ patients based on cell type [63]. Additionally, second-hit mutations with heterozygous mutations in patients’ iPSCs gave rise to RB tumors in RO, confirming Knudson’s “2-hit” hypothesis [64]. This indicates that this human Rb model can be effectively utilized to analyze the origin of Rb and the mechanism of Rb tumorigenesis, as well as screen potential therapies regarding efficacy and safety.

### Leber congenital amaurosis

LCA is a serious blinding genetic retinal disease, most of which is inherited in an autosomal recessive manner, and children usually have severe visual impairment at birth or 2 to 3 months after birth [65]. LCA affects about 1 in 81,000 people and accounts for 5% of hereditary retinal degeneration [66]. Nearly 20 pathogenic genes of LCA have been identified, most of which are specifically expressed in RPE or photoreceptor cells. The abnormality of these genes interfere with the transmission of retinal light signals to electrical signals, the metabolic cycle of vitamin A related to retinal light signaling, the differentiation and development of photoreceptor cells, and the transport and normal distribution of proteins in photoreceptor cells [67].

Patients carrying genetic changes in the gene CEP290 account for 20–25% of all LCAs and are the most common cause of LCA [68]. Retinal organoids from CEP290-LCA patients showed abnormal ciliogenesis, effectively reproducing the pathogenesis of LCA. They exhibited ciliogenesis defects, with shorter and fewer cilia in the photoreceptors compared to control ROs, indicating a tissue-specific effect of the disease-causing mutation, and correlating more widespread defects with increased disease severity [69]. NMNAT1 is a gene encoding nicotinamide adenosine dinucleotide synthetase, which is also a causative factor of LCA. By using hiPSCs and establishing NMNAT1 knock-out hiPSCs using CRISPR/cas9 technology, to reveal the role of NMNAT1 in human retinal development. The results show that NMNAT1 is dispensable for neural lineage differentiation, but is essential for retinal fate differentiation in hiPSCs. The NMNAT1-NAD-PARP1 axis may play a key role in the proper development of human retinal lineage differentiation [70]. These results all indicate that the RO model is of great significance for exploring the mechanism of LCA disease and drug screening

### X-linked juvenile retinoschisis

XLRS, named for its unique retinal division phenotype, is a relatively common early-onset degenerative disease of the central retina, with a global prevalence ranging from 1 in 5,000 to 1 in 20,000 [71]. Clinical manifestations in affected individuals encompass varying degrees of progressive central vision loss characterized by radial streaks originating from the foveal fissure and division of the inner retina in the peripheral retina [72]. RS1, a gene related to XLRS, encodes a highly conserved secreted extracellular protein named retinoschisin [73]. Retinoschisin is predominantly located on the extracellular surface of the inner segments of retinal rod and cone photoreceptors, as well as bipolar cells and two plexiform layers. It functions in cell adhesion and maintaining retinal structural integrity [74].

The researchers generated iPSCs from XLRS patients and established 3D ROs for disease research and potential treatments. The findings indicated that the XLRS-RO model replicated the key pathologies observed in patients, including the main features of XLRS, such as retinal division, deficiencies in retinal retinoschisin production, and impaired ER-Golgi transport. RS1 secretion and retinal development can be normalized by CRISPR/Cas9 gene correction, and this validated in vitro model of XLRS can be used to test and optimize treatments [75]. A total of 452 different mutations were identified in the RS1 gene, including point mutations (nonsense or missense), deletions, insertions, or splice site changes. Different forms of mutations in the *RS1* gene result in structural diversification of the RS1 protein. The c.214 G > A mutation of RS1 (p.E72K) is a frequently reported hot spot mutation. Therefore, it is important to study c.214 G > A to expand our understanding of the XLRS mechanism. Studies have shown that ROs derived from RS1 (E72K) mutant hiPSCs exhibit delayed photoreceptor development, RS1 defects, and altered spontaneous activity compared to control ROs, and can be partially salvaged by RS1 gene enhancement therapy [76]. XLRS-ROs also revealed Na/K-ATPase dysregulation due to RS1 defects and increased activity of the ERK signaling pathway. In addition, a correlated recovery of the XLRS phenotype was observed when co-cultured with control RO from healthy subjects during early differentiation [77]. In conclusion, the XLRS-RO model provides a valuable tool to elucidate the pathophysiological mechanisms of XLRS while providing insights into disease progression. Furthermore, the model provides a powerful platform for developing and optimizing targeted therapy strategies that have the potential to improve treatment outcomes for patients with XLRS.

## Conclusions

ROs are 3D cell aggregations developed on the basis of hPSCs, which can effectively simulate the developmental stages and characteristics of the human retina. These ROs demonstrate great potential in the study of retinal development, simulation of retinal diseases, and drug screening. Recent studies have further refined the methods for generating retinal organoids, enhancing the efficiency and repeatability of their generation, and notably minimizing the differences between organoids. Generally, retinal organoid technology is an evolving domain that offers new tools and platforms for retinal research, with the promise of leading to more effective treatment alternatives for patients suffering from retinal diseases in the future.

## Data Availability

Not applicable.

## Abbreviations

2D: Two-dimensional
3D: Three-dimensional
ESC: Embryonic stem cell
iPSC: induced pluripotent stem cell
ASC: Adult stem cell
PSC: Pluripotent stem cell
IRD: Inherited retinal disease
Rb: Retinoblastoma
RP: Retinitis pigmentosa
LCA: Leber congenital amaurosis
XLRS: X-linked Juvenile Retinoschisis
RO: Retinal organoid
RPE: Retinal pigment epithelium
NR: Neural retina
RGC: Retinal ganglion cell
ONL: Outer nuclear layer
OPL: Outer plexiform layer
INL: Inner core layer
OV: Optic vesicle
NPC: Neuroretinal progenitor cell
EB: Embryoid body
DDK1: Dickkopf-associated protein 1
IGF-1: Insulin-like growth factor 1
FGF2: Fibroblast growth factor 2
RPC: Retinal progenitor cell
